# Quality‐improvement program for ultrasound‐based fetal anatomy screening using large‐scale clinical audit

**DOI:** 10.1002/uog.20144

**Published:** 2019-08-05

**Authors:** M. Yaqub, B. Kelly, H. Stobart, R. Napolitano, J. A. Noble, A. T. Papageorghiou

**Affiliations:** ^1^ Institute of Biomedical Engineering, Department of Engineering Science University of Oxford Oxford UK; ^2^ Nuffield Department of Women's and Reproductive Health University of Oxford Oxford UK

**Keywords:** 2D ultrasound, clinical audit, fetal growth, pregnancy, routine anatomy screening

## Abstract

**Objective:**

A large‐scale audit and peer review of ultrasound images may improve sonographer performance, but is rarely performed consistently as it is time‐consuming and expensive. The aim of this study was to perform a large‐scale audit of routine fetal anatomy scans to assess if a full clinical audit cycle can improve clinical image‐acquisition standards.

**Methods:**

A large‐scale, clinical, retrospective audit was conducted of ultrasound images obtained during all routine anomaly scans performed from 18 + 0 to 22 + 6 weeks' gestation at a UK hospital during 2013 (Cycle 1), to build a baseline understanding of the performance of sonographers. Targeted actions were undertaken in response to the findings with the aim of improving departmental performance. A second full‐year audit was then performed of fetal anatomy ultrasound images obtained during the following year (Cycle 2). An independent pool of experienced sonographers used an online tool to assess all scans in terms of two parameters: scan completeness (i.e. were all images archived?) and image quality using objective scoring (i.e. were images of high quality?). Both were assessed in each audit at the departmental level and at the individual sonographer level. A random sample of 10% of scans was used to assess interobserver reproducibility.

**Results:**

In Cycle 1 of the audit, 103 501 ultrasound images from 6257 anomaly examinations performed by 22 sonographers were assessed; in Cycle 2, 153 557 images from 6406 scans performed by 25 sonographers were evaluated. The analysis was performed including the images obtained by the 20 sonographers who participated in both cycles. Departmental median scan completeness improved from 72% in the first year to 78% at the second assessment (*P* < 0.001); median image‐quality score for all fetal views improved from 0.83 to 0.86 (*P* < 0.001). The improvement was greatest for those sonographers who performed poorest in the first audit; with regards to scan completeness, the poorest performing 15% of sonographers in Cycle 1 improved by more than 30 percentage points, and with regards to image quality, the poorest performing 11% in Cycle 1 showed a more than 10% improvement. Interobserver repeatability of scan completeness and image‐quality scores across different fetal views were similar to those in the published literature.

**Conclusions:**

A clinical audit and a set of targeted actions helped improve sonographer scan‐acquisition completeness and scan quality. Such adherence to recommended clinical acquisition standards may increase the likelihood of correct measurement and thereby fetal growth assessment, and should allow better detection of abnormalities. As such a large‐scale audit is time consuming, further advantages would be achieved if this process could be automated. © 2018 The Authors. *Ultrasound in Obstetrics & Gynecology* published by John Wiley & Sons Ltd on behalf of the International Society of Ultrasound in Obstetrics and Gynecology.

## INTRODUCTION

A mid‐trimester fetal anatomy ultrasound scan (or ‘anomaly scan’) is offered to pregnant women in most developed countries. Clinical guidelines, such as those defined by the Fetal Anomaly Screening Programme (FASP)^1^ in the UK or the International Society of Ultrasound in Obstetrics and Gynecology (ISUOG)^2^, specify protocols for such screening. For instance, FASP recommends ultrasound image capture of six views: the head in the transventricular (TV) and the transcerebellar (TC) planes, femur length (FL), abdominal circumference (AC), the lips in the coronal view and the spine. Additionally, FASP recommends visualization (though it does not mandate image capture) of other fetal structures, such as the heart, kidneys and limbs.

Such protocols aim to guide practice of fetal ultrasonography to provide a ‘checklist’ of what imaging planes to capture. In principle, if all standard imaging planes are examined, detection of anomalies should be maximized. The quality of archived images reflects overall quality of fetal anatomical survey. Whilst this correlation is not absolute, the concern is that failure to archive images or poor image quality could support medicolegal claims.

Regular audit, peer review and quality assurance procedures are recognized to improve and sustain good practice^3^, ^4^, ^5^. However, routinely auditing images is a resource‐intensive administrative task; it is time‐consuming, tedious and takes highly trained staff away from clinical work. Consequently, a comprehensive ultrasound image audit is often undertaken only in a ‘reactive’ manner to a potential ‘screening failure’, such as in response to an unanticipated major anomaly at birth. Given the relative rarity of major anomalies, such a reactive approach is unlikely to highlight poor performance of a single operator or a department. Even when departments do perform a regular image audit, this is usually performed on a limited number of scans per sonographer. Such a selective audit is incomplete and may not provide adequate insight into sonographer performance^6^. Finally, however desirable a comprehensive image audit is, its resource‐intensive nature means that findings and feedback may seem no longer relevant to current practice^5^, ^7^.

In theory, an automated software‐based ultrasound image audit could be a solution for full (100%) clinical auditing. Putting aside technical challenges to achieve this, it is important to first ascertain whether a comprehensive image‐quality audit can improve performance.

The aim of this study was to determine whether a full clinical audit improves performance in terms of image acquisition/storage and image quality in a large maternity ultrasound department.

## METHODS

### Overview of audit cycle

This quality‐improvement study comprised a retrospective baseline audit (Cycle 1) of anonymized routine fetal anomaly scans performed over a 12‐month period in 2013 at a large teaching hospital with over 6500 births per annum. This was followed by feedback to sonographers of individual and departmental performance scores and implementation of a number of targeted interventions to improve quality of imaging. In the subsequent 12‐month period, a re‐audit (Cycle 2) using the same quality criteria was performed in order to evaluate the impact of the changes.

### Audit process

Ultrasound images and meta‐data (scan date, ultrasound machine used, sonographer ID) were extracted from the hospital database (ViewPoint, GE Medical Systems, Zipf, Austria) and anonymized to remove all patient identifiable information.

Images were audited by 12 experienced sonographers, currently practicing in different hospitals in the UK, using a standard audit pro forma including a custom‐built easy‐to‐use online interface created by Intelligent Ultrasound Ltd (Milton Park, Oxfordshire, UK). This facilitated the auditing of scan completeness (assessment of whether a scan had the complete minimum set of required images) and image quality (assessment of recorded images against a scoring system).

Before commencing the study, all assessors participated in a training day to ensure familiarity with imaging protocols, quality criteria and software, and had the opportunity to practice quality assessment using a large number of case examples.

Scan completeness and image quality were measured at the level of the department and the individual sonographer for all departmental sonographers who undertook at least 30 anomaly scans in each cycle.

### Scan completeness

According to the UK FASP guidelines^1^, a complete fetal anatomy examination should include images of the following six views: head in the TV plane, head in the TC plane, AC, FL, spine and face (lips) in the coronal plane. For the purposes of this audit, if at least one required image was missing from an examination, it was considered incomplete. Scan completeness was defined as the proportion of the total number of audited patient scans that were complete (i.e. all required images were obtained). View completeness was defined as the proportion of scans that had at least one image for a particular view.

### Image‐quality scoring

The quality of images of the head TV, head TC, AC, FL, face (lips) and spine views was also audited. The quality of each image was assessed against a number of criteria (Table 1) based on studies and guidelines, and reflective of the protocol^1^, ^8^, ^9^. A score for each image was computed as the sum of satisfied criteria in a view; the maximum achievable score was 7 for the head TV, head TC and spine images, 6 for the AC images and 4 for the FL and coronal face images. As the number of criteria varied according to view, each score was divided by the maximum score, to give a normalized score between 0 and 1. This allowed computation of an average image‐quality score overall and for each view. The median image‐quality score for each sonographer was also computed.

**Table 1:** Criteria for quality assessment of ultrasound images of head transventricular (TV), head transcerebellar (TC), abdomen, femur, spine and coronal face views

Head TV	Head TC	Abdomen	Femur	Spine	Coronal face
Symmetrical plane with midline echo of falx dividing skull	Symmetrical plane with midline echo of falx dividing skull	Stomach bubble visible	Both ends of ossified diaphysis clear	Magnification at least 30% of screen	Upper lip visible
Cavum septum visible one‐third of way along midline falx	Cavum septum visible one‐third of way along midline falx	Umbilical vein one‐third of way along anteroposterior diameter, at level of portal sinus	Angle of insonation 45–90°	Continuity intact and posterior skin edge visible	Two nostrils visible
Cerebellum not visible	Cerebellum visible	Circular plane	Magnification at least 30% of screen	Alignment of vertebrae visible	Two lip angles visible
Posterior ventricle visible at level of atrium	Cisterna magna visible	Kidneys not visible	Calipers placed on clear ends of diaphysis	Amniotic fluid visible beyond skin	Adequate magnification
Magnification at least 30% of screen	Magnification at least 30% of screen	Magnification at least 30% of screen		Lower (sacrum) visible	
Head circumference calipers placed appropriately on outer parts of skull	Calipers placed correctly on outer limits of cerebellar hemispheres	Abdominal circumference calipers placed appropriately on outer parts of abdomen		Middle (thoracic/lumbar) visible	
Calipers measuring lateral ventricles at level of atrium	Calipers placed correctly on outer limits of cisterna magna			Upper (cervical/thoracic) visible	

### Cycle 1: feedback and quality‐improvement measures

Findings from the first cycle of the audit relating to performance of the department as a whole were fed back to the sonography team. An individualized written report was also generated for each of the sonographers, who were given the opportunity to discuss any concerns or training needs. Following this, a number of actions and interventions were agreed and undertaken, including the production and dissemination of updated and formalized anomaly scan departmental protocol, the production for each scan room of aide memoire posters of the FASP base image menu (six views) required for storage, the introduction of guidance on image storage as well as a cardiac scanning protocol with specific hands‐on training sessions, and providing supported practice for individuals with specific training needs identified in Cycle 1 of the audit.

To assess the effect of quality‐improvement interventions on the performance of sonographers in the department, all anomaly scans acquired in 2014 were audited (follow‐up audit) following the same procedures used to analyze the 2013 data.

### Measuring interobserver agreement

In order to measure agreement, 10% of the audited baseline and follow‐up images were selected by computer randomization and assessed by two independent reviewers. Agreement in scan completeness was measured as the percentage of agreed images for each view (i.e. if Reviewer 1 and Reviewer 2 agreed on the view type) and a kappa statistic was calculated. The ultrasound views for which reviewers had the highest disagreement were investigated. Variability in image‐quality assessment was evaluated by comparing overall image scores. The variability for each criterion in all views was assessed to investigate which criteria contributed most to the overall variability of reviewers.

### Statistical analysis

The chi‐squared test was used to assess whether changes in scan completeness between the two cycles were statistically significant. For image‐quality scoring, paired comparisons of the normalized score (from 0 to 1) were performed using the Wilcoxon matched‐pairs signed‐ranks test, as normality tests suggested evidence of a non‐normal distribution. Two‐sided *t*‐tests are reported and the significance level was set at < 0.05. Analysis was performed using Stata 15 (StataCorp LLC, College Station, TX, USA).

## RESULTS

**Figure 1:**
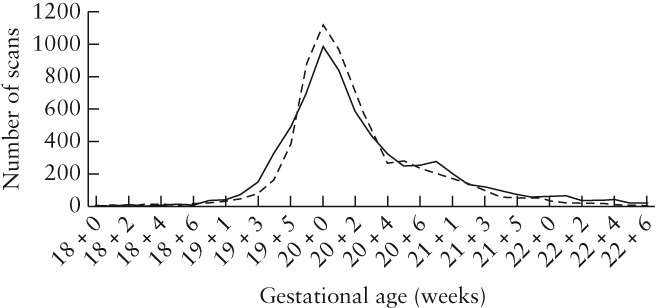
Frequency of fetal anatomical ultrasound examinations performed in a large maternity ultrasound department in 2013 (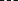) and 2014 (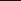), according to gestational age.

In Cycle 1, 103 501 images from 6257 anomaly examinations performed by 22 sonographers were assessed. Sonographers had varying working patterns (full time, part time or locums). The median number of scans per sonographer was 237 and the median number of audited images per scan was 16. In Cycle 2, 153 557 images were evaluated from 6406 anomaly examinations performed by 25 sonographers, of whom 20 also performed examinations in Cycle 1. The median number of scans per sonographer in Cycle 2 was 189 and the median number of audited images per scan was 24. All scans were performed between 18 + 0 and 22 + 6 weeks of gestation (Figure 1). Scans were acquired using a GE Voluson E6, GE Voluson E8, GE Voluson 730 (GE Medical Systems) and Hitachi Aloka ProSound Alpha 10 (Twinsburg, OH, USA) machines, none of which had automatic caliper placement.

### Scan‐completeness audit

**Figure 2:**
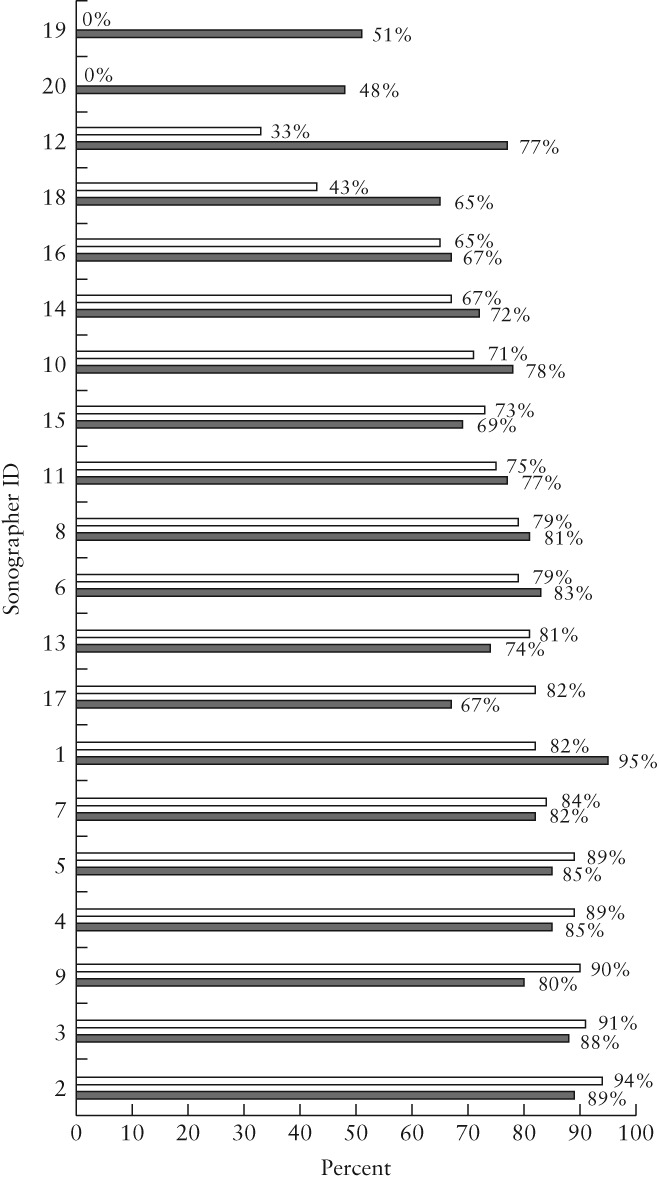
Scan completeness for fetal anatomical ultrasound examinations performed by 20 sonographers, in baseline (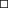) and follow‐up (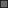) audits.

Overall scan completeness in the baseline audit was 72%, which improved to 78% in the follow‐up audit (χ^2^ = 25.99, *P* < 0.001) with improved completeness for all views. Table 2 summarizes the overall scan completeness and completeness per view in the baseline and follow‐up audits. Figure 2 shows scan completeness for all included sonographers (*n* = 20) who acquired at least 30 scans in each year. For example, sonographer 1 undertook 138 scans in Cycle 1, of which 113 were complete. The scan‐completeness audit demonstrated that the improvement was particularly noticeable for those sonographers who had the lowest completeness in the baseline audit. Sonographers whose scan completeness in Cycle 1 was less than 50% had a mean scan completeness improvement from 19.0% to 60.3% in Cycle 2. While this could be purely due to ‘regression to the mean’, this is unlikely given the consistency of improvements seen.

**Table 2:** Overall and per‐view scan completeness and quality score in baseline and follow‐up audits of fetal anatomical ultrasound examinations

View	Baseline audit	Follow‐up audit	Difference (%)*
Completeness (%)			
Head TV	89.0	92.8	+3.9
Head TC	87.4	90.7	+3.4
Abdomen	90.9	93.3	+2.4
Femur	89.9	92.9	+3.0
Spine	84.7	91.0	+6.4
Coronal face	83.5	88.9	+5.5
Overall completeness†	72.4	77.8	+5.3
Quality score			
Head TV	0.81	0.88	+0.07
Head TC	0.84	0.90	+0.07
Abdomen	0.86	0.87	+0.01
Femur	0.85	0.89	+0.04
Spine	0.68	0.77	+0.09
Coronal face	0.60	0.68	+0.08
Median overall quality score	0.83	0.86	+0.03

* Difference = (Follow‐up – Baseline).

† Proportion of complete scans for all views.

TC, transcerebellar; TV, transventricular.

### Image‐quality audit

**Figure 3:**
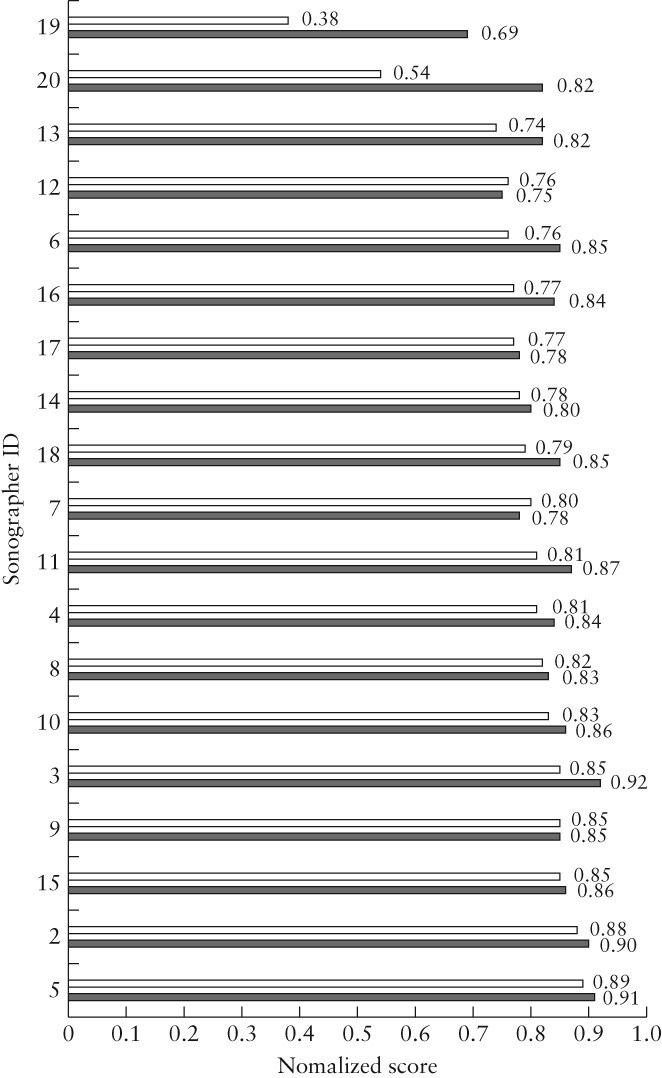
Normalized image‐quality score for fetal anatomical ultrasound examinations performed by 19 sonographers, in baseline (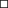) and follow‐up (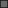) audits.

One sonographer was excluded from the image‐quality analysis due to having insufficient images for assessment in Cycle 1. For the remaining 19 sonographers who acquired at least 30 scans in each year, the median image‐quality score (for all images in all views) improved from 0.83 (interquartile range (IQR), 0.69–0.89) in 2013 to 0.86 (IQR, 0.76–0.91) in 2014 (*P* < 0.001; Table 2). The median normalized image‐quality scores for these 19 sonographers demonstrate that the greatest improvement was obtained for those with the lowest baseline audit score (Figure 3). For the nine sonographers with normalized image‐quality score < 0.80 in Cycle 1, the average quality score improved from 0.70 to 0.80 in Cycle 2, while those who had good image‐quality score at baseline performed similarly in the follow‐up audit.

### Interobserver agreement

The overall interobserver agreement between two reviewers for scan completeness was 95.1%. The agreement varied slightly between the different views, as shown by the head TV and spine images having the least agreement between sonographers (92.1% and 93.7%, respectively; Table 3). The overall mean interobserver agreement for assessing image quality was 82.7%. Table 4 shows the interobserver agreement for quality score for each view and each criterion.

**Table 3:** Interobserver agreement of 20 sonographers for assessment of fetal anatomical ultrasound scan completeness

View	Agreement (%)
Head TV	92.1
Head TC	96.5
Abdomen	96.5
Femur	98.8
Spine	93.7
Coronal face	96.8
Overall	95.1

TC, transcerebellar; TV, transventricular.

**Table 4:** Interobserver agreement in assessment of quality criteria of ultrasound images of six standard views

View/criterion	Agreement (%)
Head TV	87.0
Ventricle calipers	75.8
Head circumference calipers	82.0
Symmetrical circular brain	84.2
Cavum septum visible	86.1
Ventricles visible	88.8
Magnification	92.5
No cerebellum	99.3
Head TC	88.0
Cavum septum visible	79.5
Calipers cisterna magna	81.4
Symmetrical circular brain	88.4
Magnification	88.9
Calipers cerebellar diameter	89.2
Cerebellum visible	93.4
Cisterna magna visible	95.0
Abdomen	83.6
Abdominal circumference calipers	69.4
Umbilical vein visible	77.5
Circular	83.4
Magnification	86.5
No kidney	90.1
Stomach visible	94.4
Femur	82.2
Magnification	68.0
Femur length calipers	78.2
Clear femoral ends	88.1
Angle of insonation	94.3
Spine	75.4
Amniotic fluid visible beyond the skin	69.1
Magnification	73.2
Continuity intact and posterior skin edge visible	73.2
Alignment of vertebrae visible	74.5
Upper (cervical/ thoracic) visible	78.1
Lower (sacrum) visible	78.4
Middle (thoracic/lumbar) visible	81.3
Coronal face	80.0
Magnification	72.1
Upper lip visible	79.5
Two lip angles visible	81.2
Two nostrils visible	87.0
Overall agreement	82.7

Criteria for each view are sorted in ascending order of agreement.

TC, transcerebellar; TV, transventricular.

### Audit time

The mean reviewing time per image was 24 ± 20 s, but this varied between views, as seen in the high SD. The mean time for review per scan was 8.4 ± 6.1 min for assessment of both completeness (mean, 2.0 ± 1.2 min) and grading of gradable images (6.4 ± 5.5 min). Overall, the assessment of Cycle 1 took approximately 876 h while Cycle 2 took about 897 h.

## DISCUSSION

In this study, we assessed the effect of a full audit cycle involving large‐scale fetal anomaly scan audit on the performance of sonographers. In this audit, fetal scans were assessed manually by experts to evaluate scan completeness and image quality. A full‐year baseline audit was performed to establish the sonographers' baseline performance. Actions were implemented in the department in the following year and a second follow‐up audit was performed. We found that both scan completeness and image quality improved significantly between the audit cycles.

Although the improvement was by a relatively small margin as a department, Cycle 2 demonstrated large improvements for those sonographers who performed least well in the first audit cycle. Sonographers whose scan completeness in the baseline audit was less than 50% had a mean scan completeness improvement from 19.0% to 62.3% in the follow‐up audit, including two sonographers (sonographers 19 and 20) who had 0% scan completeness in the baseline audit. Similarly, normalized image‐quality score for sonographers whose baseline image‐quality score was less than 0.80 improved from a mean of 0.70 to 0.80 in the follow‐up audit. In contrast, sonographers who had good scan completeness and image quality at baseline performed similarly in the follow‐up audit.

Reduction in variation in practice and sustaining good practice are both recognized factors in upholding patient safety^10^, ^11^; this has also been demonstrated in fetal ultrasound. Although not evaluated as part of this study, the observed individual improvements could enhance overall screening performance of an imaging department. It is important to note that the finding of improvement in scan completeness was observed not only overall but in each individual anatomical view, and that the overall scan completeness is lower than view completeness for each view, due to the rigorous way in which we defined scan completeness.

Interobserver agreement was high for the assessment of scan completeness; it was lowest for the head TV view, which may be because the original local protocol was unclear as to whether the head TV or transthalamic (TT) view should be stored, and therefore some recorded head views were in the TT view. Although TV and TT planes are very close to each other^12^, this could introduce some disagreement between reviewers. The interobserver agreement was higher for completeness compared with assessment of image quality. This is likely to be due to the more complex nature of image‐quality assessment, which includes assessing several criteria per view. It is also evident that assessment of caliper placement is not as reproducible as other criteria (Table 4). For instance, the agreement on the placement of AC calipers was less than 70% and the worst among other abdominal criteria.

One of the key strengths of this study is that the entire scan output of a single department was assessed, removing any potential biases in selection of examinations for review and offering the most comprehensive assessment of both departmental and individual performance. A limitation of this approach is that it is very labor intensive. Although it is possible that a retrospective audit of a small number (e.g. 10–20 cases) of consecutive scans would suffice, this would provide only limited understanding of individual and departmental performance. Another option, self‐scoring, in which sonographers appraise their own images, has been shown previously to be feasible^13^. However, this self‐scoring was undertaken within a framework of additional, independent scoring; it is not known whether this would be of benefit in the absence of such external review^13^.

In our study, independent visual assessment was performed by experienced sonographers. This was based on an objective, criteria‐based scoring system that was shown to have a high degree of reproducibility for image scoring. High reproducibility (kappa, 92.7) for image scoring methods shown in a previous study^8^ was also seen in the current study, and confirms the high level of training of the reviewers conducting the audit. Despite this, the assessment of placement of calipers in the four biometry views typically had a low agreement between reviewers (Table 4); the significant contribution of caliper placement to variability is in keeping with the findings of previous studies, such as that of Sarris *et al*.^14^. In addition, although the femur is a long straight object, there was significant disagreement between reviewers when assessing magnification of the femur images. In contrast, criteria that assessed if an object is not visible on an image, for example, no cerebellum in the head TV view, had high agreement between reviewers.

One potential limitation of this study was that the two audit cycles were back‐to‐back. This was to allow seamless observation of practice over time. It is highly likely that, had we allowed a period of time to pass before re‐audit to ensure that changes in practice in response to Cycle 1 were more embedded, greater improvements would have been seen. Given the described audit process, it is possible that some of the observed improvements in performance may have related more to sonographer awareness of ongoing audit and knowledge of the specific criteria used to evaluate performance. However, a longer study period could have been subject to confounding due to other unmeasured changes. Another limitation is that we assessed only six views, based on the recommendations for practice in the UK; we, of course, recognize that other scanning protocols suggest a larger number of images to be stored. However, the improvements seen in this smaller number of views should also apply. Although imaging of cardiac views was introduced in 2014, this was on the basis of best practice and they were not included in the analysis as they were not assessed in Cycle 1.

In conclusion, we have shown that large‐scale clinical audit, coupled with implementation of targeted changes and feedback to sonographers, can lead to improvements in image quality on the mid‐trimester anomaly scan, in terms of both completeness of scans and image quality. However, while quality improvement is possible, such comprehensive manual audit in a high‐throughput clinical setting is a very labor‐intensive process and this would be a major barrier for implementation in routine practice. Ongoing work on automated image analysis^15^ and further research into automated image recognition would open up the possibility of more rapid audit processes, and the current work provides evidence that this could be effective.
